# Decoding Mastitis in Small Rodent Pets: Pathophysiology, Antimicrobial Resistance Profiles, and Future Directions in Treatment Strategies

**DOI:** 10.3390/antibiotics14101000

**Published:** 2025-10-09

**Authors:** Raul Alexandru Pop, Iosif Vasiu, Cosmina-Andreea Dejescu, Piera Anna Martino, Marco Wochnik, Roman Dąbrowski

**Affiliations:** 1 Department of Obstetrics and Gynecology, Faculty of Veterinary Medicine, University of Agricultural Science and Veterinary Medicine, 3-5 Mănăştur Street, 400372 Cluj-Napoca, Romania; alexandru.pop@usamvcluj.ro; 2 Department of Anaesthesiology and Surgery, Faculty of Veterinary Medicine, University of Agricultural Sciences and Veterinary Medicine, 3-5 Mănăştur Street, 400372 Cluj-Napoca, Romania; cosmina.dejescu@usamvcluj.ro; 3 One Health Unit, Department of Biomedical, Surgical and Dental Sciences, University of Milan, 20133 Milan, Italy; piera.martino@unimi.it; 4 Department and Clinic of Animal Reproduction, Faculty of Veterinary Medicine, University of Life Sciences, Głęboka 30, 20-612 Lublin, Poland; marco.wochnik@up.lublin.pl (M.W.); roman.dabrowski@up.lublin.pl (R.D.)

**Keywords:** rodent, mammary gland, mastitis, pets

## Abstract

**Background:** Mastitis is a condition affecting various animals, including hamsters, gerbils, mice, and rats. While these species are frequently used as models for studying mammary gland infections in biomedical research, there is limited understanding of their natural occurrence, epidemiology, and management. This review examines the anatomy, causes, clinical signs, pathology, and treatment of mastitis in small pet rodents, highlighting existing knowledge gaps and emphasizing the need for further research. To the best of the author’s knowledge, this is the first review focusing on mastitis in small pet rodents, underlining the importance of maintaining mammary gland health in these pets.

## 1. Introduction

Mastitis is the inflammation of glandular tissue, a condition that affects all mammals [1]. Among the included mammals are those from the order Rodentia that are kept as pets, such as the Campbell’s dwarf hamster (*Phodopus campbelli*), the Russian hamster (*Phodopus sungorus*), the golden Syrian hamster (*Mesocricetus auratus*), the Mongolian gerbil (*Meriones unguiculatus*), the house mouse (*Mus musculus*), and the brown rat (*Rattus norvegicus*).

Furthermore, it is diagnosed equally in primiparous and multiparous dams, simultaneously affecting one or multiple mammary glands [2,3]. Usually, infections are ascending and have a bacterial etiology. However, even though small rodents are often used in human medicine as models for experimental mammary gland infections [4,5,6,7,8,9,10], the presence of mastitis after natural infections in these animals is rare [2]. Unfortunately, studies regarding the prevalence or epidemiology of mastitis in this category of pets are limited or absent.

On the contrary, a great deal of information is available regarding mastitis in farm animals. Mastitis is one of the most common diseases affecting dairy cows worldwide. It accounts for approximately 60–70% of all antimicrobials administered on dairy farms, and the disease leads to severe direct and indirect economic losses [11,12]. Although the microorganisms responsible may be shared with the causative agents of mastitis in small domestic rodents, the pathogenic mechanisms, epidemiology (little known in pet rodents), antibiotic treatments, and above all, the economic repercussions are so different that it is not possible to compare the forms in different mammalian species adequately. This review aims to highlight the current state of knowledge, as depicted in the literature.

## 2. Materials and Methods

The data search was performed using Google Scholar and PubMed databases. Keywords used included “rat,” “mice,” “hamster,” “gerbil,” “small rodent pets,” “mastitis,” “mammary gland,” “infection,” “inflammation,” and “pathology.” Various combinations and strategies were employed to locate relevant data. No date restrictions were applied, and only papers in English were included. Additionally, the reference lists of articles and reports were examined to find relevant publications that might not be indexed. Hard copies of articles unavailable at the University of Agricultural Sciences and Veterinary Medicine of Cluj-Napoca, Romania, were obtained through interlibrary loan services from other European libraries. All relevant articles were thoroughly reviewed in full text by the authors (IV, CAD, PAM). When disagreements arose, resolutions were achieved through mutual consent. This study encompasses all pertinent information from case reports, reviews, and book chapters.

## 3. A Small Glimpse into Small Rodent Mammary Gland Anatomy

Similar morphological characteristics of mammary glands exist among mice, rats, and gerbils [13]. Thus, the mammary glands exhibit a tubuloalveolar compound morphology, consisting of branching tubular ducts that end in secretory glandular acini, also called alveoli in rats. Each lobule comprises groups of acini. Multiple lobules combine to form a large lobe. Moreover, the fundamental milk-producing unit of the *mamma* is the terminal duct lobular unit (TDLU) [14] (Table 1).

The TDLU drains into the intralobular and extralobular terminal ducts, collects ducts, and empties into the lactiferous ducts through the nipples’ ostia. The TDLU appears in mice during gestation, but in female rats, it seems to develop upon sexual maturity. In mice, the mammary gland tree of a non-lactating female terminates in a blind ductile [14].

The mammary gland acini are lined with one or two layers of cuboidal epithelial cells, which serve as functional secretory cells during lactation. Additionally, the appearance of the mammary gland changes throughout the estrous cycle. During proestrus and estrus in rodents, epithelial cell proliferation at the outer edges of the gland regresses during metestrus and diestrus [14].

However, the mammary gland epithelium is more abundant during the first 14 days of gestation. The acini are lined with simple cuboidal epithelium, while the terminal portion of the ducts is lined with squamous epithelium. Secretory epithelium partially replaces the adipose tissue [13]. In mice, rats [14], gerbils [13], and hamsters [18], there is only one lactiferous duct in each mamma, which drains milk into the nipple [14] (Table 1).

**Figure 1:**
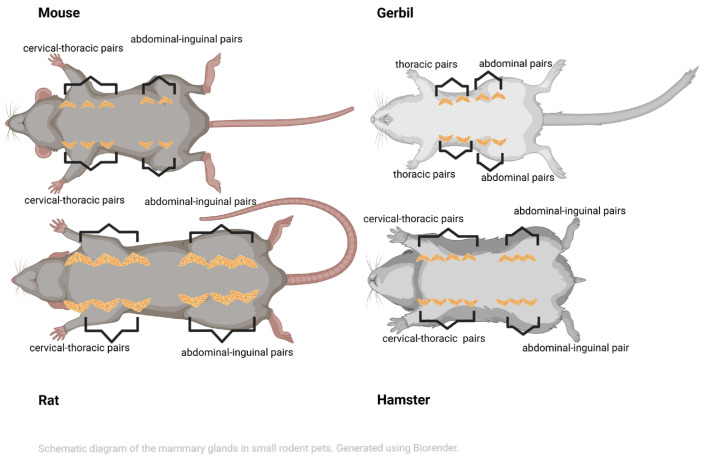
Schematic view showing the layout of the mammary glands in small pet rodents. In hamsters, up to seven pairs of mammary glands can be found; in gerbils, there are four pairs; in rats, there are six pairs; and in mice, there are five pairs (created in BioRender. Vasiu, I. (2025) https://BioRender.com/bxkhm3h).

In female rats [15] and mice [14], six symmetrical pairs of mammary glands are located along the ventral milk line between the neck (including the elbow) and the inguinal area—three cervical–thoracic and three abdominal–inguinal in rats, and five pairs—three cervical–thoracic and two abdominal–inguinal—in mice (Figure 1). An axillary portion extends dorsally to the shoulder [14,15]. Moreover, the mammary gland fat pads are extensive in both species, wrapping from the ventrum to the cranially interscapular area and from the lateral flanks caudally to the perineal region [14].

In female gerbils, four symmetrical pairs of mammary glands (i.e., two thoracic and two inguinal) are situated along the ventral milk line between the thorax and the inguinal area [13,18] (Figure 1).

However, in female hamsters, six to seven symmetrical pairs of mammary glands—specifically, four cervical–thoracic and two abdominal–inguinal—are located along the ventral milk line, extending from the thorax to the inguinal area [16,18]. In contrast, the Russian hamster only has four pairs of mammary glands [16] (Figure 1).

## 4. Definition, Etiology, and Pathogenesis

Mastitis is primarily diagnosed in lactating individuals, particularly in the inguinal pair, due to their proximity to the perineal region, which facilitates the transfer of pathogenic bacteria and even septicemia in nursing pups. Furthermore, mammary gland infections are mainly spread through galactogenous or percutaneous means (i.e., ascending infections) or, less commonly, via hematogenous or endotoxic routes (i.e., descending infections) [3,19,20,21].

It occurs after pathogenic bacteria invade the mammary tissue and multiply, which activates the immune system, thus generating proinflammatory mediators, increasing vascular permeability, and causing inflammation of the local mammary gland [17].

The bacterial strains responsible for mammary gland infections in these rodents are detailed in Table 2, with the Staphylococcaceae and Pasteurellaceae families being the most predominant. Additionally, the presence of *Rodentibacter pneumotropicus* (formerly *Pasteurella pneumotropica*), *Escherichia coli,* and *Klebsiella pneumoniae* strains may contribute to the development of focal mammary gland abscesses [2,3,22,23].

Weak immune systems, trauma (such as bites from hamster pups, which have sharp teeth that emerge early), or stress from environmental factors like dirty bedding, poor sanitation, and substandard husbandry practices can all lead to infections that promote the spread and growth of bacteria, causing mastitis. This weakens the outer layer, increasing its vulnerability to disease [29,30].

Recent research on rats shows that heat stress damages the integrity of the mammary gland epithelium, making it easier for pathogens to invade and cause damage during experimental mastitis induced by *K. pneumoniae*. This occurs by weakening the barrier and triggering an inflammatory response, which can lead to more severe mastitis [21]. The change in epithelial barrier permeability may affect the migration of white blood cells from the blood to the infected mammary tissue. Animals under heat stress begin blood redistribution, increasing peripheral blood flow to help dissipate heat [21]. Additionally, in heat-stressed rats, heat waves, worsened by climate change, can increase the occurrence and severity of mastitis by impairing rodents’ immune defenses and epithelial barriers [31,32,33].

The blood–milk barrier (BMB) is a specific structure that plays an important role in protecting mammary gland function in mammals. Increased permeability of the BMB leads to a significant influx of somatic cells, especially polymorphonuclear leukocytes (PMNs) in the mammary gland, worsening the inflammation [34,35,36].

Interleukins (ILs) are proinflammatory cytokines released by activated macrophages that promote inflammatory reactions and play a key role in the body’s response to infection. Myeloperoxidase (MPO) activity serves as a biomarker of tissue infiltration by neutrophils and is directly linked to the number of infiltrating cells early in the inflammatory process [6,36].

During lipopolysaccharide (LPS)-induced mastitis in mice, activated macrophages migrate from the mammary interstitium to the alveolar space, leading to the production of various biochemical markers. After tissue injury, interferons, ILs (interleukin-beta, IL-1β; interleukin-6, IL-6; and tumor necrosis factor, TNF-α) and MPO, are released into the bloodstream [4,5,6,7,8,9,36]. Other inflammatory enzymes like Cyclooxygenase-2 inhibitors (COX-2), or Inducible Nitric Oxide Synthase (iNOS), which produces nitric oxide in response to inflammatory stimuli, are also elevated during mammary gland infections [35,36].

Interestingly, during the acute phase of inflammatory reactions in mice, IL-6 plays the most significant role among the three proinflammatory cytokines [6]. Nevertheless, the increased levels of ILs and MPO activity during LPS-induced mastitis reflect neutrophil infiltration in the mammary tissues [4,5,6,7,8,9,36].

However, when mice developed mastitis caused by Coagulase-negative staphylococci (CNS; *Staphylococcus aureus*, *S*. *chromogenes*, *S*. *fleurettii*), the inoculated *S*. *aureus* produced IL-6 and IL-1β but not TNF-α. In general, CNS-inoculated glands showed a weak cytokine host response, yet they still triggered local IL-1β production. Unlike other CNS strains, *S*. *chromogenes* from the teat apex triggered a more varied IL-1β response, with a stronger local reaction in several mice [27]. Additionally, a recent study found that levels of IL-6, IL-1β, TNF-α, and MPO were all increased in mice that developed mastitis due to *S*. *aureus* [28].

Inoculating mice with *Clostridium tyrobutyricum* protected against *S*. *aureus*-induced mastitis by activating the vagus nerve to release oxytocin (OT), which then inhibits the NF-κB signaling pathway by decreasing the production of oxytocin receptor (OTR) proteins. By lowering these protein levels, OT helps reduce the inflammation caused by *S*. *aureus*, thus decreasing circulating levels of IL and MPO activity, decreasing damage to the mammary gland, and regulating the permeability of the BMB [34].

The Granulocyte colony-stimulating factor (G-CSF) is a growth factor that promotes the development and activity of mature neutrophils. Moreover, chemokines such as CXCL1 and CCL2 attract macrophages and monocytes; therefore, the induction of these biomarkers may suggest a role for these cells in the host response [23].

Following *E. coli*-induced mastitis in mice, G-CSF, IL-6, CXCL1, and CCL2 are released into the peritoneal fluid. Elevated levels of these substances are also found in blood plasma, indicating a systemic inflammatory response caused by *E*. *coli*. Most likely, the production of these compounds acts as a protective mechanism for the host by promoting the recruitment of neutrophils to the infection site. Levels of G-CSF and CXCL1 may be elevated in severe, systemic bacterial infections [23].

Certainly, the mechanisms of mastitis in small rodent pets are much more complex than described here. Therefore, future research should focus on developing frameworks to clarify these complex etiopathological mechanisms. Case reports and cohort studies of lactating small rodents should be conducted during the periparturient period and screened for natural clinical and subclinical mastitis cases.

## 5. Clinical Signs

Generally, local signs of inflammation, such as hot, red, engorged, or painful mammary glands, indicate episodes of mastitis in small rodents [2]. However, in the golden Syrian hamster (GSH), ten days postpartum, during a mastitis episode caused by an *E*. *coli* infection, hypertrophy of the entire mammary chain was reported, displaying a yellowish-gray coloration with red central miliary areas and firm, removable subcutaneous mammary gland nodules in the inguinal region. In addition to the local signs, the presence of vaginal discharge was also noted, accompanied by the mortality of the entire litter [3] (Table 3).

In an experimentally induced mastitis with co-infection of *E. coli* and *S. aureus* in mice [23], symptoms such as weakness, lethargy, muscle tremors, lack of response to stimuli, and keratoconjunctivitis sicca (KCS) were observed, along with purulent epiphora and a greenish film on the cornea, followed by the development of intestinal imbalances [23]. The intake of toxic milk, agalactia, septicemia, and enteritis, along with the failure of dams to nurse, significantly contributes to a high rate of neonatal mortality [3].

Despite the limited data on clinical mastitis episodes in pet rodents, additional research is essential to enhance our understanding of mammary gland inflammation in these species. Studies should aim to clearly delineate the clinical distinctions between clinical mastitis episodes (both acute and chronic), particularly for chronic cases where symptoms are harder to recognize and can be misinterpreted as various neoplastic nodules. It is also important to differentiate between septic episodes (either acute or chronic) and non-septic conditions (such as mammary congestion and galactostasis).

According to the authors’ knowledge, there is a lack of published data on subclinical mastitis in small rodent pets. Since no obvious signs of local or general mammary gland inflammation are expected in dams, issues like failure of offspring to thrive, lack of weight gain in newborns, and fetal or neonatal mortality might still occur. Future research should focus on describing such cases in all small rodent pets (Table 3).

Data on laboratory changes during mastitis episodes is also limited; however, normochromic microcytic anemia with anisocytosis, poikilocytosis, and polychromasia, along with moderate neutrophilia and lymphopenia, has been reported in the GSH during the *E. coli* mastitis episode. Moreover, proteinuria and moderate hemoglobinuria may also be present [3].

In white star rats, compared to C-reactive protein (CRP), the levels of haptoglobin (Hp) and fibrinogen (Fbgn) increase significantly during experimental inflammation [37]. In other experimental studies involving mice and rats, during mastitis-induced episodes, increased levels of IL-1β, IL-6, TNF-α cytokines, and MPO were observed (see Section 4) [2,6,7,8,9,21,28,35,36,37] (Table 3).

Recognizing the challenges in collecting blood, especially milk samples from these animals, further research is crucial to assess variations in blood and milk concerning mammary gland infections or injuries. Studies on cats and dogs [38,39] indicate that a complete blood count (CBC) should be performed whenever mastitis is suspected. In small rodents, an increase in white blood cell (WBC) counts is anticipated alongside anemia, with signs such as toxic or band neutrophils, phagocytosing macrophages, and foamy cells observed in blood smears [40]. The milk pH is expected to be alkaline, reflecting a positive acute phase protein (APP) response, both in milk and the bloodstream [37]. Future research should focus on elucidating the laboratory changes linked to the different types of clinical or subclinical cases of mastitis, whether septic or non-septic.

Unfortunately, the prognosis remains challenging. Many cases of mastitis go unrecognized, and there are few published reports. Severe complications for both dams and their pups pose serious risks, contributing to high mortality rates.

## 6. Histopathology

To the authors’ best knowledge, data regarding histopathologic changes in natural mammary gland inflammations in small rodent pets is minimal. In a case of clinical mastitis caused by an infection with *E*. *coli* in a GSH, discrete or confluent multiple foci of coagulation necrosis, accompanied by a neutrophilic infiltrate, edema, small amounts of fibrous tissue, degenerated neutrophils, and scattered macrophages, were found around the necrotic areas from the mammary gland nodular lesions [3]. In addition to the mammary gland lesions, pathologic changes in the uterus and liver were also noted in both natural and experimental mammary gland infections caused by *E. coli* and *S. aureus*, respectively [3,23].

However, more data exists on histopathological changes in small rodents used as experimental models. Experimentally co-infecting mice with *E*. *coli* and *S*. *aureus* results in distinct, firm mammary gland nodules. These nodules are pinkish-yellow and surrounded by a consistently thin, pink, sandy capsule with a glandular structure. Multiple cystic cavities containing numerous neutrophils, bacterial forms, and a yellowish caseous exudate were also observed. This type of infection caused significant neutrophil recruitment into the peritoneal cavity, followed by slightly elevated eosinophil numbers, a modest increase in lymphocytes, and no changes in macrophages or mast cells [23].

In *K*. *pneumoniae*-induced mastitis in rats, there was acinar hemorrhage, acinar shrinkage, more severe hyperemia, and neutrophil infiltration [21]. In LPS-induced mouse mastitis, the inflammatory mammary lesions included interstitial edema, hyperemia, milk stasis, thickened walls of the mammary alveoli with numerous inflammatory cells such as neutrophils and macrophages, and acinar necrosis with neutrophil infiltration, alternating with healthy glandular tissue. The mammary alveoli were more hyperemic and thicker than in control glands, with infiltration of inflammatory cells into the alveolar lumen [4,5,6,7,8,9,36].

In another study, LPS-induced chronic mastitis in mice causes a severe inflammatory response, with the mammary gland alveoli and ducts heavily infiltrated by mononuclear cells, including lymphocytes, macrophages, and plasma cells. Severe fibrosis occurs in the chronically inflamed tissue, accompanied by a lack of milk production in the secretory units [19].

Studies on mice with CNS-induced mastitis reveal a substantial increase in neutrophils in the mammary gland, with *S*. *aureus* causing the most severe inflammation. This is marked by a high number of invading neutrophils and red blood cells in the interstitium [27]. Additionally, mammary gland tissue may show larger acini, thicker acinar walls, colloid secretions in the alveolar cavity, and a more complete honeycomb structure, along with acinar wall hyperplasia [28]. Extensive necrotic areas are also observed [26].

Additional research is essential to understand the distinct pathological changes in the mammary gland associated with each clinical type of mastitis. These changes can be influenced by anatomical sites, morphological characteristics, physiological conditions (such as lactating or non-lactating status), and species-specific traits.

## 7. Medical Management

Mastitis treatment in small rodents primarily involves antibiotic therapy, such as enrofloxacin, chloramphenicol, or trimethoprim, ideally chosen based on culture and sensitivity results, while ensuring that the gastrointestinal tract remains unaffected (Table 4). Penicillin could be used and acts as an antibacterial by inhibiting bacterial cell wall synthesis, a process crucial for bacterial survival. They block the formation of peptidoglycan, the main component of the wall. Gram-positive bacteria (i.e., *S. aureus*) are the main target of penicillin. Chloramphenicol is a protein synthesis inhibitor that binds to the 50S subunit of bacterial ribosomes with bacteriostatic activity. One significant class of broad-spectrum antibacterial drugs is fluoroquinolones. Quinolones block the actions of two enzymes necessary for bacterial survival: DNA *gyrase* and *topoisomerase* IV [41,42]. Sulfonamide antibiotics specifically inhibit a crucial enzyme in the folic acid metabolic pathway. The metabolic pathway enzyme *dihydropteroate synthase* (DHPS) is the target of sulfonamide antibiotics. However, *dihydrofolate reductase* (DHFR), another enzyme in the same pathway, is the target of trimethoprim antibiotics [42].

However, penicillin is contraindicated in certain species, such as hamsters and gerbils, as it can easily cause dysbiosis, which may be fatal. Furthermore, fluoroquinolones should be avoided in young mammals due to the potential risk of cartilage defects occurring during their growth phase, especially in mice and rats. Specific analgesics, including meloxicam, should not be used in dehydrated animals experiencing hypovolemic shock, coagulation disorders, or gastrointestinal complications [43,44].

Additionally, based on their experiences with small rodents, the authors find the results of susceptibility testing discouraging (personal observations), as a significant percentage of tested samples exhibit antibiotic resistance. Therefore, creating preventive strategies is crucial to minimizing inappropriate antibiotic use and preventing spillover. The challenges of administering medication to these small pets, as well as the inconsistent antibiotic dosages noted in various textbooks [43,44], further underscore this issue. The authors emphasize the need for additional research to establish clear dosing guidelines.

Moreover, supportive care remains essential. To alleviate pain, the use of opioids and nonsteroidal anti-inflammatory drugs (NSAIDs) like meloxicam can be administered to lessen inflammation and tissue swelling. Warm Epsom salt compresses can also help reduce inflammation and enhance local blood circulation in the affected areas. Severe cases of mastitis may necessitate debridement to remove necrotic and damaged tissue [2,30].

Severe complications can arise during mastitis episodes, and given the current treatment limitations for small rodent populations, clinicians often encounter a lack of alternatives. Future research must focus on clarifying treatment options and scenarios, as the distinction between successfully treating or saving these small pets is marginal.

Experimentally, it has been shown that the administration of curcumin extracts (*Curcuma longa*) [4], baicalein (*Scutellaria biacalensis*) [5], indirubin (Indigo feratinctoria) [6], salidroside (*Rhodiola rosea*) [8] or emodin (*Rheum rhabarbarum*) [7], houttuyfonate salt [9], maslinic acid [36], or the *Chinese pasqueflower* (*Pulsatilla chinesis*; PCE) [28], in lactating doe mice, can improve, or even protect, the mammary gland against local inflammatory processes.

Other studies [34] have shown that supplements containing probiotics, such as *C*. *tyrobutyricum*, originating from the gastrointestinal tract of these rodents can enhance the body’s protective mechanisms against the effects of mastitis [34]. Orally administered *Lactobacillus reuteri* also reduced the impact of local infection in *S. aureus*-induced mastitis in mice, as evidenced by a significant increase in the relative abundance of *L. reuteri* in both the gut and the mammary gland [47]. Additionally, using maslinic acid in mice with mastitis may help stabilize the gut flora, potentially playing an anti-inflammatory role by boosting probiotics and reducing the presence of harmful bacteria [36].

Moreover, to prevent the transfer of bacterial strains, toxins, and antibiotics to suckling pups via milk, it is recommended to separate pups from dams affected by mastitis. Therefore, the pups have to be fed artificially; placing them next to surrogate mothers, although it may increase their chances of survival, may lead to the spread of mastitis to the surrogate female and cannibalization of the newly introduced litter. However, to be more readily accepted by the surrogate doe, vanilla essence (*Vanilla planifolia*) can be used, thus reducing the risk of the foster doe rejecting orphans [2,30].

Unfortunately, as a rule, hamster pups that do not suckle from their mother usually succumb. Nevertheless, hand nursing is possible, especially in orphan hamster pups, via feeding tubes, syringes, or in the form of eye drops (watch out for the development of aspiration bronchopneumonia) with kitten milk replacements, goat’s milk, or other homemade formulas. Nevertheless, this does not necessarily increase the survival rate of the pups. Thus, the increased risk of high neonatal mortality does not diminish consistently [2,30].

## 8. Antibiotic Resistance in Small Companion Rodents with Mastitis: Current Evidence, Clinical Implications, and Management Strategies

Veterinary medicine is increasingly concerned about antibiotic resistance in small pet rodents with mastitis. In general, one of the main causes of bacterial population resistance in treated animals has been found to be antibacterial treatments. Furthermore, people may be exposed to animal-derived antibiotic-resistant bacteria (ARB) and antibiotic-resistant genes (ARGs) directly or indirectly through environmental contamination. Moreover, ARGs can move horizontally through mobile genetic elements, increasing the likelihood that human gastrointestinal coliforms will include ARGs from milk bacteria [48,49,50].

Data from ruminants, which share some bacterial pathogens with rodents, show that they are highly resistant to amoxicillin (50%), streptomycin (42.8%), tetracycline (40.4%), lincomycin (39%), and erythromycin (33.8%). This presents a more significant concern for mammalian mastitis overall [48,49].

This issue significantly impacts public health and clinical care. The growing resistance of these infections to antibiotics makes it more challenging to develop effective treatment plans and raises the risk of treatment failure, chronic illness, and zoonotic transmission. However, there is currently very little information available about the prevalence and effects of antibiotic resistance in cases of mastitis in pet rodents.

### 8.1. Patterns of Antibiotic Resistance in S. aureus-Induced Mastitis

Small pet rodents with mastitis show antibiotic resistance profiles similar to those seen in other mammals worldwide. They are particularly resistant to antibiotics commonly used in treatment. Evidence indicates that hamsters are resistant to penicillin [51,52,53]. MRSA *S. aureus* isolates in mice demonstrate high resistance to clindamycin (up to 79%) and moderate resistance to trimethoprim-sulfamethoxazole (35%) [53]. Most of these MRSA strains carry the *mecA* gene, which confers resistance to all beta-lactam antibiotics. For *S. aureus*, the resistance to penicillin is primarily based on the production of β-lactamase enzymes, which hydrolyze the β-lactam ring and make the antibiotic useless; many *S. aureus* strains can survive in the presence of methicillin because PBP2a, which is encoded by the *mecA* gene, decreases the bacteria’s affinity for β-lactam antibiotics. Although uncommon, vancomycin resistance in *S. aureus* has been documented, and the primary cause of this resistance is the acquisition of genes from vancomycin-resistant enterococci (VRE). The acquisition of the chloramphenicol–florfenicol resistance *cfr* gene is one mechanism by which S. aureus develops resistance to chloramphenicol. Fluoroquinolone resistance in *Staphylococcus* can arise by antibiotic efflux. One crucial step is the overexpression of the chromosomally encoded efflux pumps (NorA, NorB, and NorC). Topoisomerase mutations have also been identified as a contributing factor to the emergence of fluoroquinolone resistance [54].

There is limited information on bacterial resistance in rats; however, environmental factors like heat stress can worsen mastitis, which may promote the growth of resistant bacteria [31,48]. Many people use antibiotics such as enrofloxacin, chloramphenicol, and trimethoprim, but resistance patterns vary; therefore, susceptibility testing is recommended to choose the most effective treatment [48,49].

### 8.2. Focus on Gram-Negative Bacteria as an Etiological Agent of Mastitis in Small Companion Animals

Multiple factors, like pathogenesis, anatomical particularities, and environmental factors, complicate the understanding of bacterial mastitis in small pet rodents. Feces, urine, or contaminated bedding can promote the indirect spread of resistant bacteria and their genes into the environment. These microbes can survive and be transmitted to other animals or humans, especially in households with pet rats. Resistant germs may remain in dust and on surfaces for long periods, increasing the risk of human exposure. This risk is significantly higher with multidrug-resistant Gram-negative bacteria such as extended-spectrum beta-lactamase (ESBL)-producing *E. coli* and *K. pneumoniae*, which have been found in both clinical and subclinical mastitis cases in rats and other small mammals [10,55,56,57,58]. These organisms are particularly concerning since they can transfer genes between different species and even across genera, further increasing the potential for zoonotic transmission. Additionally, shared living spaces where people and dogs coexist can be breeding grounds for resistant bacteria.

Gram-negative bacteria, especially *E*. *coli* and *K*. *pneumoniae*, are increasingly recognized as causes of mastitis in small rodent pets, including hamsters, mice, rats, and gerbils. However, Gram-negative infections can significantly impact health and may lead to severe mastitis, resulting in high mortality. The outer membrane of Gram-negative bacteria contains LPS, which is primarily responsible for their virulence. LPS is a potent endotoxin that triggers a strong local and systemic inflammatory response. This cascade involves the release of proinflammatory cytokines, including IL-1β, IL-6, and TNF-α, which rapidly attract neutrophils, damage tissue, and, in severe cases, lead to septicemia [21,59].

One of the significant challenges in treating Gram-negative mastitis in rodents is the growing antibiotic resistance. Studies on both laboratory rodents and small ruminants (whose mastitis pathogens are often similar to those of rodents) have shown that *E. coli* and *Klebsiella* isolates are highly resistant to tetracyclines, beta-lactams, and, to a lesser extent, aminoglycosides [60,61]. The four main resistance mechanisms for Gram-negative bacteria include the following: remodeling of outer membrane porins and biofilm formation; modification of antibiotic target sites in the bacterium, such as Lipid A, 16s rRNA, and PBPs; increased efflux pump action to transport antibiotics out of the cell actively; and the activity of beta-lactamases and aminoglycoside-modifying enzymes to inactivate/modify antibiotics. The paper by Gauba & Rahman (2023) describes the mechanisms by which Gram-negative bacteria develop antibiotic resistance in depth [62].

ESBLs, produced by genes like *bla_CTX-M_* and *bla_TEM_*, further complicate therapy because these enzymes confer resistance to most penicillin and cephalosporins [60,61]. Additionally, efflux pumps and mutations in target sites reduce drug effectiveness, increasing the risk of disease transmission from animals to humans.

Because of these challenges, the best way to treat Gram-negative mastitis in small rodents is with a multifaceted, evidence-based approach. Culture and antimicrobial susceptibility testing are highly recommended to help select the appropriate treatment, as choosing antibiotics based solely on assumptions is becoming less reliable. Third-generation cephalosporins, including ceftiofur, and, in some cases, aminoglycosides, may be options, but their use must be balanced against the risk of nephrotoxicity and species-specific contraindications [63].

### 8.3. Alternative Treatments to the Use of Antibiotics

Because antibiotics do not always work, treating mastitis in small pet rodents requires a multifaceted approach. It is not recommended to rely solely on empirical antibiotic therapy. Instead, culture and sensitivity testing should be performed whenever possible to ensure that the treatment is targeted to the resistance profile of the isolated organism [53].

In mouse models, experimental treatments such as bacteriophages, probiotics, and plant extracts have shown promise in reducing bacterial load and inflammation, which could decrease reliance on traditional antibiotics [53].

High host specificity, quick phage isolation, the ability to combine different bacteriophages (phage cocktails) for increased effectiveness, and co-administration with antibiotics to lower antibiotic resistance are just a few benefits of bacteriophages. For example, bacteriophage therapy is effective against MRSA-induced mastitis in mice, making it a targeted alternative to antibiotics [53,64].

Researchers have also explored the anti-inflammatory and antibacterial properties of probiotics, such as *Limosilactobacillus reuteri DSM 17938*, a type of probiotic that can colonize the skin, breast milk, urinary tract, and gastrointestinal tract [65].

The effectiveness of plant-based substances has also been evaluated. Berberine, a quaternary amine with anti-inflammatory, immunomodulatory, antibacterial, antifungal, antiviral, and antiparasitic properties, was tested in conjunction with extracts of *Cyperus rotundus*, which possess anti-inflammatory, anticancer, immunomodulatory, analgesic, antibacterial, and antioxidant properties, in a rat mastitis model. Additionally, the effects of *Pulsatilla chinensis*, with anti-inflammatory, antioxidant, and antipathogenic properties, were also evaluated in experimentally induced mastitis of mice [28,66]. The results obtained with these plant extracts have proven promising.

### 8.4. Surveillance and Future Directions

It is vital to use antimicrobials carefully and based on evidence when treating mastitis in small rodents within a One Health (OH) framework. In acute or severe cases, empirical therapy might be needed; however, it should be used sparingly whenever possible in favor of targeted treatment guided by culture and sensitivity testing. This method not only supports recovery for the individual animal but also helps prevent the development of resistant strains that could spread to other animals or humans. When choosing antibiotics, consider species-specific contraindications (Table 4) [28,66,67,68].

The OH approach to treating mastitis in small rodents relies on research and monitoring. As indicated by the limited data in the current literature, there is an urgent need for systematic studies to determine the prevalence of mastitis in these species, its clinical features, laboratory diagnosis methods, and the most effective therapies.

Surveillance should include both clinical instances and subclinical infections, which can harbor resistant bacteria and quietly facilitate the spread of antimicrobial resistance (AMR) within and between species. The increasing presence of mastitis pathogens (Table 5) in small companion rodents that are resistant to antibiotics highlights the need for careful monitoring, prudent antibiotic use, and the development of new treatment approaches. To understand how AMR spreads in rodent populations and their environments, it is necessary to study molecular epidemiology, focusing on identifying resistance genes and their transmission pathways. We also require integrated surveillance systems that connect data from veterinary, medical, and environmental sources to detect new hazards and support risk assessment and management.

Education and communication are also crucial components of the OH approach to small rodents with mastitis and AMR. Pet owners, veterinarians, and animal caregivers must understand the risks of antibiotic misuse, the significance of cleanliness and environmental management, and the potential for resistant germs to spread to humans. Public health messages should emphasize responsible pet ownership, including regular veterinary visits, keeping pets clean, and properly disposing of pharmaceuticals and hazardous materials.

Veterinarians play a vital role in antimicrobial stewardship by guiding pet owners on the correct use of antibiotics and promoting preventive measures, such as reducing stress and maintaining good husbandry practices [69,70]. Veterinarians are urged to perform culture and sensitivity tests to ensure that antibiotic therapy is most effective, helping to prevent resistance and improve patient outcomes.

## 9. Conclusions

The overall impacts of mastitis in small pet rodents extend beyond immediate clinical and veterinary issues to include broader concerns such as public health, environmental sustainability, and global health security. The rise in AMR in rodent mastitis cases underscores the challenge of maintaining separate human, animal, and ecological health. Because these areas are connected, changes in one can influence the others. For example, better hygiene and husbandry when caring for rodents can lower the rate of mastitis and reduce the need for antibiotics. This decreases the pressure for resistance development and lessens the environmental impact of antimicrobial drugs. Conversely, using antibiotics on pets without careful consideration can worsen the global AMR epidemic, affecting human medicine, agriculture, and ecosystem health [71,72,73].

The OH approach emphasizes the need to establish and follow guidelines and standards covering all aspects of antibiotic use in pets. These should align with current frameworks for food animals and human health, as resistant germs and genes can spread across different sectors. To track the increase and spread of AMR, particularly in the context of global trade in pets and animal products, countries must collaborate and share information. Funding for research should prioritize studies that address gaps in our understanding of rodent mastitis epidemiology, causes, treatments, alternative and complementary therapies, such as probiotics and botanical extracts, along with the environmental impact of veterinary antibiotics [69,71,74].

This review provides a summary of the existing understanding of mastitis in small rodents, highlighting the information gaps on the subject. The literature could be greatly improved by investigating all facets of mastitis, including its clinical and laboratory characteristics, such as prevalence, epidemiology, symptomatology, histopathology, and laboratory tests (i.e., from milk and/or blood). Clinicians should also remember that, although mastitis is infrequent in small rodents, the complications can be severe, particularly due to the high neonatal mortality rate and the risk of female mortality.

## Figures and Tables

**Table 1:** Anatomical and histological features of mammary glands in small pet rodents (modified after [14]).

Anatomy and Histology Features					References
	Rat	Mice	Hamster	Gerbil	
Major lactiferous ducts	Single lactiferous duct or sinus forming up to 10 secondary branching ducts; single complex branching.		Single complex branching.		[13,15,16,17]
Functional glandular unit	LA unit; matures only during gestation.	TDLU; matures only during gestation.	Lobules with acini and ducts that transport milk, surrounded by fat. The ducts are lined with a luminal and basal layer of epithelial cells.	Ductal system with bilayered epithelium of outer myoepithelial cells and inner luminal cells, forming ducts and alveoli surrounded by fat.	
Premature mammary gland	No LA unit; the ducts have blunt ends.	TDLU forming.			
Mature mammary gland	Only adipose tissue with minimal connective tissue surrounding quiescent ducts.			Increase in epithelial cell height and acini diameter; the glandular epithelium replaces the adipose tissue; the collagen composition of the stroma changes. Alveolar lumens become distended with milk.	
Lactating mammary gland	Well-formed acini with intraluminal milk and nuclear atypia.				

Abbreviations: LA, lobuloalveolar; TDLU, terminal duct lobular unit.

**Table 2:** Pathogens isolated from milk of the Rodentia order (modified from [24]).

Phylum	Morphology and Gram Staining	Milk Pathogens	References
Actinobacteria	High G + C Gram-positives	*Corynebacterium bovis*	[25]
		*Trueperella pyogenes*	
		*Mycobacterium bovis*	
Mycoplasmatota		*Acholeplasma laidlawii*	
Streptococcus Firmicutes	Gram-positive bacilli	*Lactococcus lactis*	[26]
	Gram-positive cocci	*Staphylococcus aureus*	[6,23,26,27,28]
		*Staphylococcus chromogenes*	[26,27]
		*Staphylococcus epidermidis*	[25]
		*Staphyloccocus lentus*	[27]
		*Mammaliicoccus fleurettii*	[27]
		*Staphylococcus saprophyticus*	[25,27]
		*Streptococcus acidominimus*	
		*Streptococcus agalactiae*	
		*Streptococcus uberis*	
	Gram-negative cocci	*Mycoplasmopsis agalactiae*	
		*Mycoplasmopsis bovigenitalium*	
		*Mycoplasmopsis bovirhinis*	
		*Mesomycoplasma dispar*	
		*Mycoplasma mycoides*	
		*Mycoplasmopsis arginini*	
		*Mesomycoplasma ovipneumoniae*	
		*Mycoplasmopsis bovis*	
Proteobacteria	Gram-negative bacilli	*Pseudomonas aeruginosa*	
		*Mannheimia haemolytica*	
		*Rodentibacter pneumotropicus*	[2]
		*Escherichia coli*	[3,25]
		*Klebsiella pneumoniae*	[2,21]
	Gram-negative curved bacilli	*Campylobacter coli*	[25]
Fungi	Yeast-like	*Candida albicans*	
		*Candida krusei*	

**Table 3:** Clinical and laboratory changes in small pet rodents with subclinical and clinical mastitis.

	Clinical Findings	Laboratory Findings	References
Subclinical mastitis ^a^	Dams—no obvious signs of inflammation. Offspring—failure to thrive; neonatal death; loss of entire litter.	Leukocytosis, anemia, alkaline milk pH, elevated levels of APPs, increased somatic cell count in milk, and the presence of milk pathogens.	
Clinical mastitis	Dams—local signs—hypertrophy, discolored skin, nodules. Systemic signs—vaginal discharge, agalactia, septicemia, fever, weakness, tremors, KCS, purulent epiphora, and enteritis. Offspring—failure to thrive, neonatal death, and loss of the entire litter.	^a^ Milk analyses—alkaline pH, numerous degenerated neutrophils, engulfed bacteria on milk smears, and the presence of pathogenic milk microorganisms. Hematology—normochromic microcytic anemia, neutrophilia, and lymphopenia. Biochemistry—elevated levels of Hp, IL-1β, IL-6, TNF-α or MPO, proteinuria and hemoglobinuria.	[3,4,5,6,7,8,9,21,23]

Abbreviations: APPs, acute phase proteins; Fbgn, fibrinogen; Hp, haptoglobin; IL-1β, interleukin-1 beta; IL-6, interleukin-6; KCS, *keratoconjunctivitis sicca;* TNF-α, tumor necrosis factor; MPO, myeloperoxidase. ^a^ anecdotal data. Future research is necessary to evaluate the specificity and sensitivity of these parameters in lactating dams.

**Table 4:** List of drugs used in the treatment plan of small rodents diagnosed with mastitis.

Drug Class	Drugs	Dosages *
Penicillin	Amoxicillin ^a^	100–150 mg kg IM SC BID
Amphenicols	Chloramphenicol	30–50 mg/kg IV IM SC PO SID/BID
Fluoroquinolones	Enrofloxacin ^b^	5–20 mg/kg SC PO SID/BID
Sulfonamides	Trimethoprim ^c^	50–100 mg/kg PO SC SID
NSAIDs	Meloxicam ^d^	1–2 mg/kg SC PO SID
Opioid analgesic	Buprenorphine	0.01–0.05 mg/kg IM SC BID TID

Abbreviations: NSAIDs, nonsteroidal anti-inflammatory drugs; BID, two times a day; IM, intramuscularly; IV, intravenously; PO, orally; SC, subcutaneous; SID, once a day; TID, three times a day. * dosages may vary according to different textbooks. Clinicians should consult various bibliographic references before choosing the proper treatment plan [43]’ ^a^ the administration of penicillin in the hamster and gerbils is prohibited [44]; ^b^ fluoroquinolone administration is prohibited in small mammals due to reported growing cartilage defects in mice and rats [44,45,46]; ^c^ in animals with KCS, the administration of sulfonamides should be avoided, as well as in patients with adverse effects to these drugs [44]; ^d^ not to be administered to dehydrated, hypovolemic, hypotensive animals or with coagulation defects or gastrointestinal impairment. Furthermore, the pros and cons must be judiciously evaluated before administering these drugs to animals with chronic renal insufficiency. Moreover, in gestational dams, in pups under 6 weeks, and during the perioperative window, these drugs are prohibited [44].

**Table 5:** Key findings on affected species, bacterial isolates, resistance patterns, and clinical treatment options [32,43,44,45,46].

Species	Bacterial Species Isolated	Resistance Profile	Clinical Treatment
Hamster	*Staphylococcus aureus*, *Escherichia coli*, Pasteurellaceae, *Rodentibacter pneumotropicus*	Resistance to penicillin; fluoroquinolones contraindicated (growth cartilage defects); variable resistance to enrofloxacin, chloramphenicol, and trimethoprim	Enrofloxacin, chloramphenicol, trimethoprim; NSAIDs (meloxicam); warm compresses; debridement in severe cases
Mouse	*Staphylococcus aureus* (including MRSA), *Escherichia coli*, Staphylococcaceae, Pasteurellaceae	MRSA strains with *mecA* gene; high resistance to clindamycin (79%) and trimethoprim-sulfamethoxazole (35%); low resistance to linezolid and doxycycline	Bacteriophages; antibiotics based on susceptibility testing; probiotics; botanical extracts
Rat	*Klebsiella pneumoniae*, *Staphylococcus aureus*, *Escherichia coli*	Resistance data limited; concern for antibiotic resistance, especially under heat stress	Cautious antibiotic use; supportive care; experimental therapeutics under study

## Data Availability

The original contributions presented in this study are included in the article. Further inquiries can be directed to the corresponding author.
